# Experimental Study of the Impact of Glass Beads on Adhesive Joint Strength and Its Failure Mechanism

**DOI:** 10.3390/ma14227013

**Published:** 2021-11-19

**Authors:** João P. J. R. Santos, Eduardo A. S. Marques, Ricardo J. C. Carbas, Frida Gilbert, Lucas F. M. da Silva

**Affiliations:** 1 Instituto de Ciência e Inovação em Engenharia Mecânica e Engenharia Industrial (INEGI), 4200-465 Porto, Portugal; up201605813@fe.up.pt (J.P.J.R.S.); carbas@fe.up.pt (R.J.C.C.); 2 ArcelorMittal Global R&D, 60160 Montataire, France; frida.gilbert@arcelormittal.com; 3 Departamento de Engenharia Mecânica, Faculdade de Engenharia (FEUP), Universidade do Porto, 4200-465 Porto, Portugal; lucas@fe.up.pt

**Keywords:** adhesive bonding, automotive industry, glass beads, material characterization

## Abstract

The use of modern structural adhesives provides a lightweight, practical, and high strength joining methodology, which is increasingly being adopted in the automotive and aeronautical sectors, among many others. However, the strict mechanical performance standards that must be met in these applications require a constant search for ways of improving the adhesives’ behavior, which has led to the growing use of reinforcements as a way of improving the capabilities of bonded joints. The aim of this work was, thus, to analyze how the addition of inorganic fillers to the adhesive layer affects a joint’s strength and its failure mechanism. To this end, single lap joint specimens with mild steel and high strength steel substrates were tested, at quasi-static speeds, and with different amounts of glass microspheres reinforcing two different structural adhesives. The experimental results indicated that the addition of glass particles reduced the joint performance for both substrates under study. Furthermore, the failure pattern was found to evolve from adhesive failure to a cohesive type of failure as the amount of glass particles present in the adhesive was increased.

## 1. Introduction

As highly technological industries, including those in the automotive and the aeronautical fields, increase their usage of adhesively bonded joints, the demands placed on the mechanical performance of these connections have also greatly increased. Consequently, a set of innovative techniques and solutions have been proposed, in an attempt to improve the adhesives’ performance [1,2]. Some of these more recent approaches are based on the hybrid joint concept, which consists of a combination between adhesive joints and other conventional joining methods, the search for an optimal adhesive layer thickness, or even modifications to the adherends’ surface roughness and surface preparation to improve adhesion [3,4].

However, many of these techniques are complex and expensive, which may discourage their practical use. Taking this into account, the introduction of reinforcements into the adhesive layer has grown as a practical alternative in the search for better performing adhesive joints. It should be noted that the addition of reinforcements to the adhesive forms a composite material, since it turns into a combination of two or more materials [3,5]. Many authors have drawn attention to the fact that the nature of the fracture surface of the bonded joint is of utmost importance to the performance and safety of the joint. The work of Bond et al. is strict in demanding a rigorous analysis of the fracture surface in aerostructures, matching the level of detail applied to metallic failures. They see this as a fundamental step to ensure that the technology is sound at the design, certification, and production stages [6]. However, failure modes can be highly dependent on factors beyond the level of adhesion. Hasheminia et al. studied the fracture mechanism of joints with similar and dissimilar materials with composite substrates, observing adhesive failure and delamination failure. When adhesive failure was found, boundary lines were also found, associated with some plastic deformation of the adhesive [7]. A similar work was carried out by Kanani for hybrid joints, where the authors found a correlation between the stiffness mismatch of the adherends and the failure mode of the joint; this generated a large shear stress distribution near the interface with the adherend with lower stiffness. This was found to mostly depend on the nature of the adherends and less on the type of adhesive tested [8].

Several important experimental studies have been carried out on the effects of adding reinforcements to the adhesive layer. Barbosa et al. [9] tested multiple specimen configurations with different amounts of cork, between 0.5% and 5%, in the adhesive layer. This experimental study showed that, for 1% of cork particles, the adhesive became more ductile, and this led to higher joint strength. However, increasing the amount of particles to 2% was found to lead to a noticeably worse mechanical behavior, since the particles started acting as defects.

Kinloch et al. [10] studied the effects of the addition of nanosilica particles to a rubber toughened adhesive. These particles were found to improve the adhesive’s toughness and shear strength, for quantities between 1% and 8% in weight. The formation of stress fields around the particles and near the crack tip was found to contribute to additional plastic deformation of the adhesive matrix.

Imanaka et al. carried out work on the fracture toughness of DCB specimens bonded with silica filled adhesives. The fracture toughness was found to increase with particle size and also with the degree of interfacial strength between the silica and the adhesive matrix. A mixture of crack pinning and crack blunting was determined to be responsible for this effect [11].

A work of Zhang et al. was dedicated to improving the performance of epoxy joints with the increase of CNT contents up to 0.75%. In this case, the CNTs were found to be able to minimize the deleterious effects of larger adhesive thicknesses on joint strength, while ensuring that the failure mode remained cohesive in nature [12].

Furthermore, da Silva et al. [13] focused on the influence of cork microparticles in the failure mode of single-lap joints. This led to the conclusions that, by increasing the size and amount of the cork microparticles, the failure mode changes from adhesive failure to a cohesive failure. In addition, it was observed that the failure load only decreases when 5% of 75–125 nm and 250–500 nm particles are introduced.

Similarly, Ayatollahi et al. studied two different toughening particles (silica nanoparticles and multi-walled carbon nanotubes) for adhesive reinforcement. Both particles led to modest strength improvements, but the fractography results indicated that adding nanoparticles caused a change from an adhesive failure mode towards a dominant cohesive failure mode. The authors interpreted this change as the result of improved adhesion between the adhesive and adherends [14].

Hunter et al. [15] investigated how the presence of glass microspheres in an adhesive affected the mechanical adhesion of single-lap joints with fiber-reinforced polymers; concluding that the use of these particles has different effects, depending on how fast the adhesive is cured. For a slow-curing adhesive, the joints’ strength decreased for the 3% and the 10% weight concentrations of glass microspheres, when compared to the neat adhesive’s configuration. For the fast-curing epoxy adhesive, the joint’s strength increased for both concentrations, when compared to the joints with neat adhesive. The work of Chimeni et al. investigated the effect of glass bead size and content on the properties of virgin and recycled polyethylene (LDPE) composites. A morphological analysis demonstrated a high amount of particle pull out. The authors postulated that the weak level of interfacial adhesion limits the level of mechanical reinforcement, especially under tensile loadings, with a decrease in performance with increasing glass bead content [16]. Vahthrus et al. compared the performance of hydrophobic silica aerogel powder and hollow glass microspheres (HGM) fillers for the improvement of the thermal insulating properties of an epoxy material. Their work showed that the bonding strength of the resultant epoxy composites increased with the addition of aerogel and decreased when the glass spheres were used as the filler material. It was demonstrated that, while these glass spheres increase the insulating potential of epoxy resins, there is an accompanying decrease in the mechanical properties of the resulting composite [17]. 

In the present work, the impact of reinforcing adhesives with glass beads was investigated; searching for changes in the joint performance and, importantly, in the mechanism and location of the failure. This change is of great importance, since some industrial users prefer failure to occur in the central portion of the adhesive layer and not near the substrate–adhesive interfaces, even if the performance is slightly lowered. To that end, hollow glass beads were added to two epoxy adhesives, considering different amounts of particles. In order to assess joint strength, as well as the failure modes, single lap joint specimens were manufactured and then tested. The corresponding fracture surfaces were studied, using SEM analysis to assess the damage state of the glass beads and ascertain the damage mechanisms that took place.

## 2. Experimental Details

### 2.1. Materials

The adhesives analyzed in this work will be referred to as Adhesive A and Adhesive B. These adhesives are both one component crash-resistant adhesives, based on an epoxy chemistry, which are used with the aim of increasing crash performance and body stiffness in automotive construction. Both cure at a temperature stage of 180 °C for 30 min.

**Figure 1:**
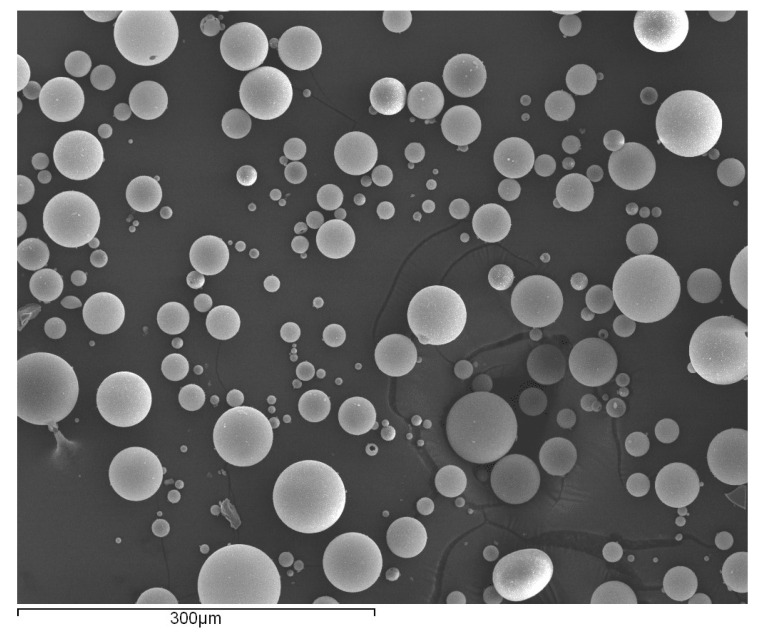
SEM micrograph of glass microspheres.

The hollow glass beads used (Figure 1) consist of thin-walled hollow spheres made of soda-lime-borosilicate glass, with a white powder-like appearance. Their size and density can slightly vary, and they are known to be lightweight and have a high strength-to-density ratio and a significant isostatic crush strength [18,19]. Glass beads were selected for this work since they are already used for control of the adhesive thickness in many applications; thus opening the possibility for a dual use. This is also a quite inexpensive material, representing an added cost of less than 0.01 USD per joint.

Table 1 lists the main properties of the glass beads used.

### 2.2. Specimen Fabrication

For this experimental study, two different single lap joint geometries were analyzed, mainly differing in the material of the adherend and its dimensions.

**Figure 2:**
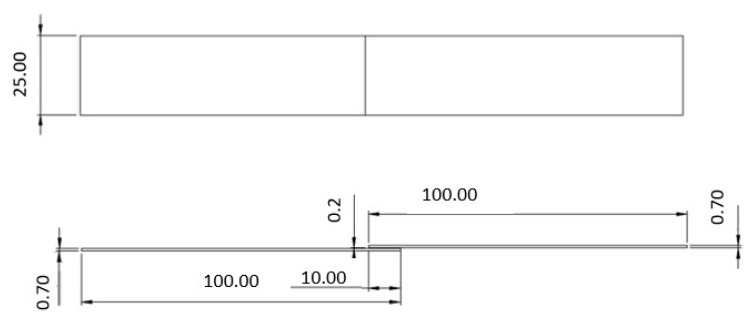
Representation of the geometry of the single lap joint specimens using mild steel substrates, all dimensions in mm.

One of the geometries that was analyzed is represented in Figure 2. For this single lap joint configuration, an adherend composed of mild steel was used.

The adherend surfaces to be bonded were degreased with acetone, with the objective of cleaning the surfaces and ensuring the proper removal of any kind of contaminants, dust, or oils.

**Figure 3:**
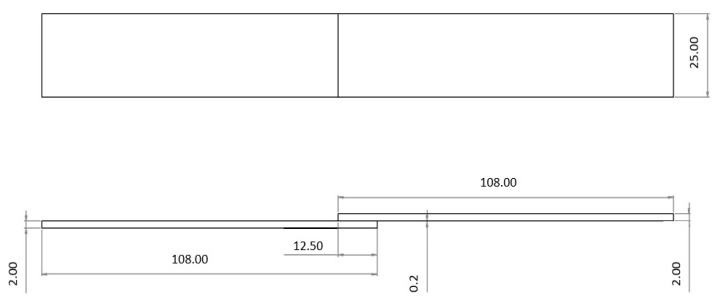
Representation of the geometry of the single lap joint specimens using high strength steel substrates, all dimensions are in mm.

The other SLJ specimen configuration used an adherend manufactured from a high strength steel (DIN C65, heat treated), with a Young’s modulus of 210 GPa and a Poisson’s ratio of 0.3. This configuration was selected in order to avoid plastic deformation of the adherend. The geometry and dimensions of the SLJ are detailed in Figure 3. Please note that the bonded area is slightly different, as both the mild steel and the high strength could only be supplied in these specific sizes. 

The adherend surfaces to be bonded were first subjected to a surface treatment. The surfaces were grit blasted and then degreased with acetone. The main goal of this surface treatment procedure was to clean the surfaces and ensure the proper removal of weak layers, contaminants, dust, or oils.

The manufacturing of the single lap joint specimens was achieved with the use of a custom designed mold. 

The addition of glass beads into the adhesive layer was achieved by mixing them in the pre-heated epoxy adhesive, in order to facilitate the distribution of the particles along the adhesive matrix. This was performed using a centrifuge mixing machine, SpeedMixer DAC 150^TM^ (Hauschild, Hamm, Germany) for 40 s, increasing gradually from a 500 r/min to a 3000 r/min rotation speed within the first 10 s. The rotation speed was then maintained at 3000 r/min for the remaining time. This procedure was adopted for all quantities of glass microsphere under study. After curing, the specimens were removed from the mold, and the excess of adhesive which flowed away from the bonded area was manually eliminated, with the use of a file. Four specimens were manufactured (and tested) for each configuration. Specimens bonded with adhesive A were tested in the neat state and with 5%, 10%, and 15% of added particles in volume. Specimens bonded with adhesive B were limited to the neat state, and 5% and 10% of added particles in volume. As will be shown in the result section, 15% of added particles in volume was not pursued for adhesive B, since this was found to be above the amount required to trigger the transition in failure mode.

### 2.3. Testing Procedures

An INSTRON 3367 universal testing machine was used to test the single lap joints. This machine is equipped with a load cell of 30 kN (INSTRON, Norwood, MA, USA).

The tests were conducted at a quasi-static speed of 1 mm·min^−1^. For each test, the load–displacement (*P-δ*) curve was registered until failure occurred. Three specimens were tested for each condition. A high-speed camera was used in order to visualize the crack propagation path along the adhesive layer.

### 2.4. Scanning Electron Microscopy Analysis

Scanning electron microscope (SEM) analyses were performed utilizing a JEOL JSM 6301F/Oxford INCA Energy 350/Gatan Alto 2500 microscope (JEOL, Tokyo, Japan). This equipment also includes an X-ray photoelectron spectroscopy analyzer (XPS). The combination of SEM and XPS allowed the analysis of the single lap joint specimens fracture surfaces, the chemical composition of the particles being studied, as well as their level of damage.

## 3. Experimental Results

### 3.1. Single Lap Joint Tests with High Strength Steel Substrates

It is important to mention that the neat configuration of Adhesive A already had 5% in volume of hollow glass beads, since this is the manufacturer supplied state of this adhesive.

**Figure 4:**
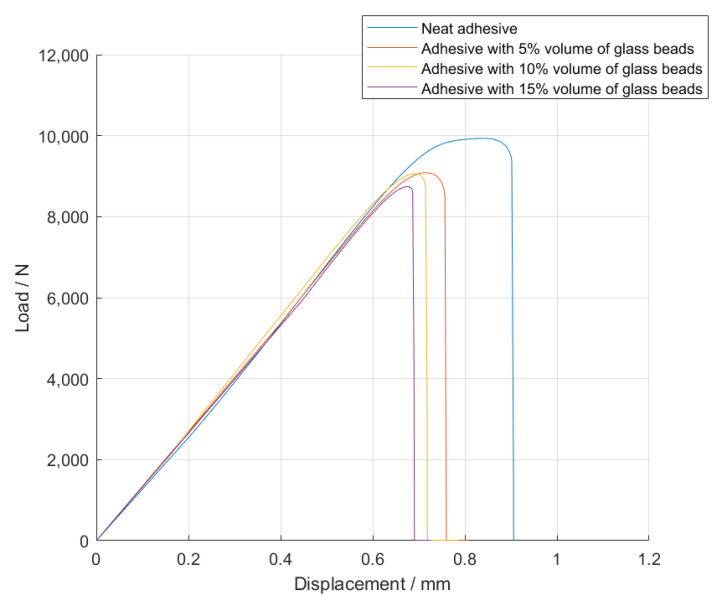
Representative *P-δ* curves for the single lap joint tests of Adhesive A with neat adhesive, and the addition of 5%, 10%, and 15% volume of hollow glass beads.

The *P-δ* curves obtained for the single lap joint specimens with high strength steel are shown in Figure 4, for Adhesive A under quasi-static conditions.

**Figure 5:**
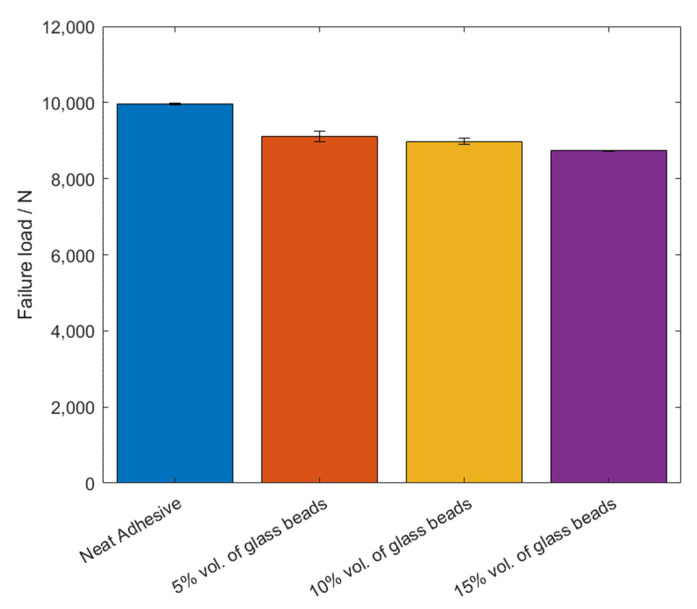
The failure load values, *P_max_*, of Adhesive A for the different percentage volumes of hollow glass beads added.

**Figure 6:**
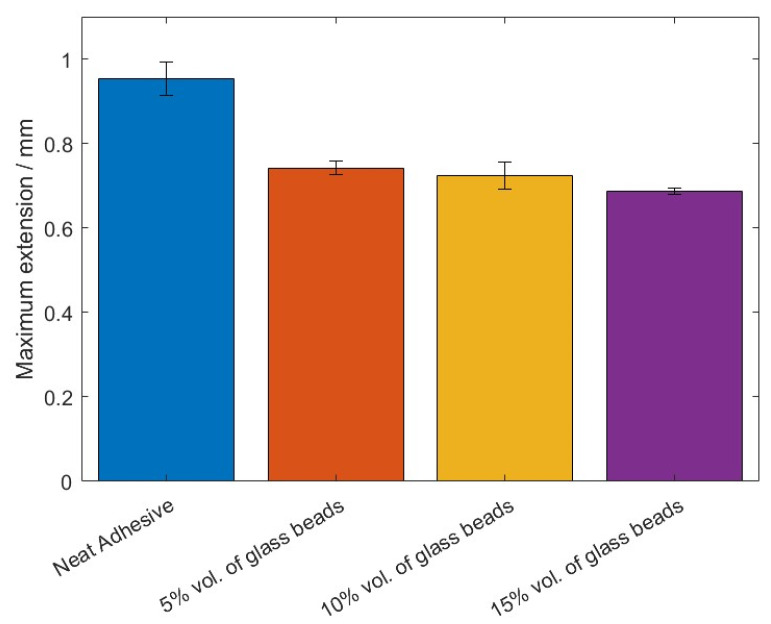
The maximum extension values, *δ_max_*, of Adhesive A for the different percentage volumes of hollow glass beads added.

The values of the failure load, *P_max_*, and the maximum extension, *δ_max_*, can be observed in Figure 5 and Figure 6, for the different percentage volumes of hollow glass beads added.

Based on the *P-δ* curve results and the mean values of maximum extension, it can be concluded that adding particles to the adhesive layer had a negative impact on the joint performance, since not only the area below the curves decreased, but the maximum extension was also reduced. Therefore, for the single lap joints with high strength steel substrates, the presence of glass beads had a negative impact on the mechanical behavior of the bonded joints. Regarding the failure load, the effects caused by the presence of the particles can be considered negligible.

**Figure 7:**
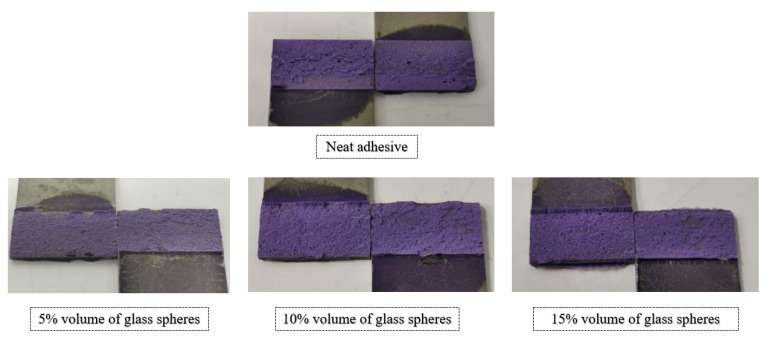
SLJ fracture surfaces with Adhesive A as a function of the amount of added hollow glass beads that were employed.

With respect to the failure patterns, Figure 7 illustrates the fracture surfaces of the SLJs for the different percentage volumes of hollow glass beads added that were evaluated.

These images show that there was clearly a cohesive failure for all the four configurations under study, which means that, for this single lap joint geometry, the presence of glass beads does not affect the failure mode of the bonded joints.

**Figure 8:**
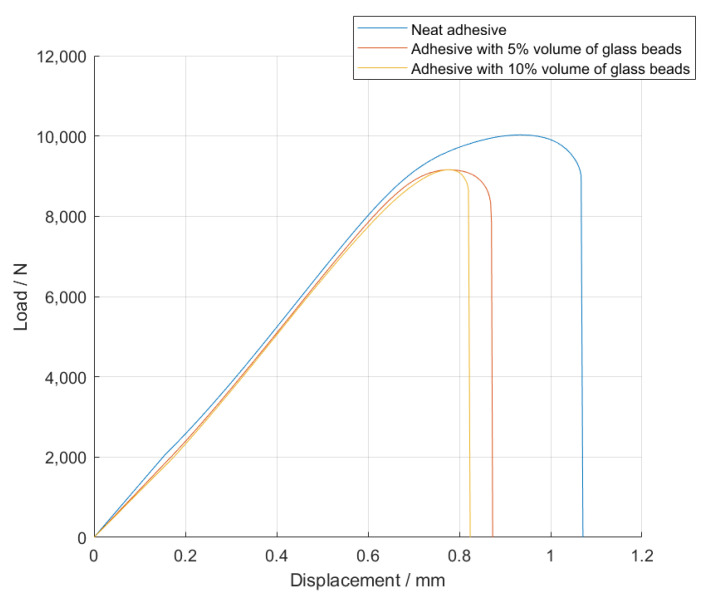
Representative *P-δ* curves for the single lap joint tests of Adhesive B with neat adhesive, and the addition of 5% and 10% volume of hollow glass beads.

The *P-δ* curves associated with the SLJs with Adhesive B are represented in Figure 8.

**Figure 9:**
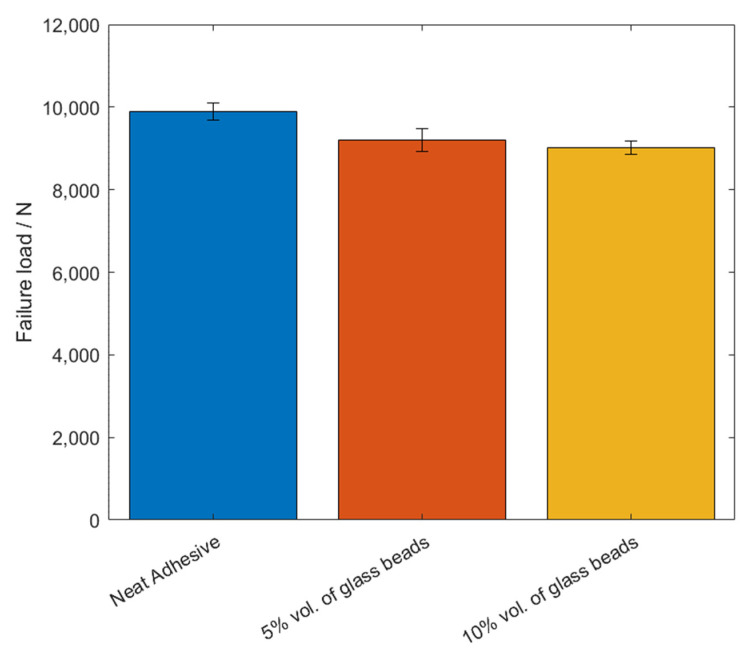
The failure load values, *P_max_*, of Adhesive B for the different percentage volumes of hollow glass beads added.

**Figure 10:**
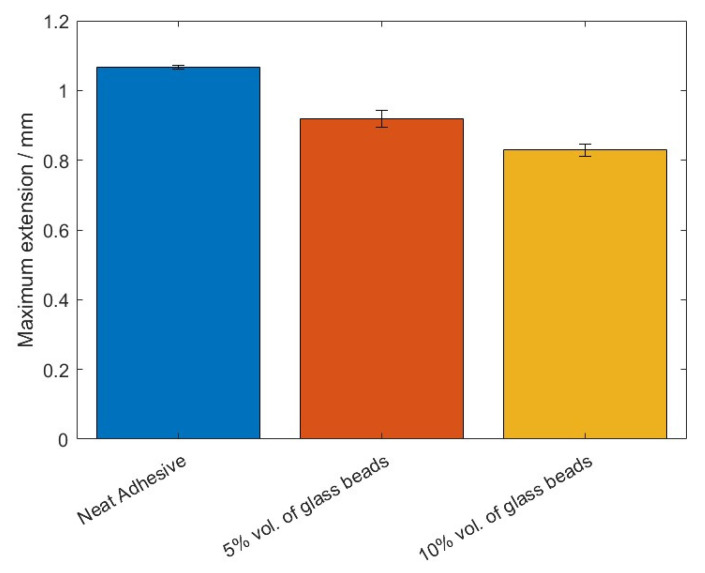
The maximum extension values, *δ_max_*, of Adhesive B for the different percentage volumes of hollow glass beads added.

The failure load, *P_max_*, and the maximum extension, *δ_max_*, mean values, as a function of the added of hollow glass beads amounts are displayed in Figure 9 and Figure 10.

For this adhesive, and similarly to what was reported for Adhesive A, the introduction of glass particles into the adhesive layer worsened the mechanical behavior of the bonded joints, since the area of the curves represented in Figure 8 decreases with an increasing amount of particles. Moreover, the maximum extension, *δ_max_*, significantly decreases, and the failure load, *P_max_*, does not show a significant correlation to the amount of glass particles.

**Figure 11:**
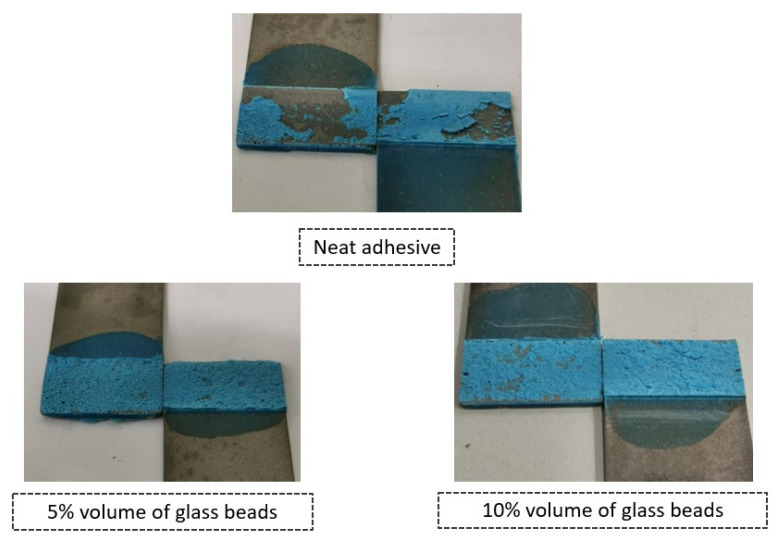
SLJs fracture surfaces of Adhesive B as a function of the amount of added hollow glass beads that were employed.

Figure 11 illustrates the fracture surfaces of the SLJs for the different percentage volumes of hollow glass beads that were evaluated.

The fracture surfaces clearly show that, by introducing glass particles into the adhesive layer, the failure evolves from an adhesive failure towards a cohesive one. This is a consequence of the local stress concentrations formed on the periphery of the glass particles; the weak links in the adhesive layer. Subsequently, cracks will initiate near those points, rather than at the ends of the bonded joints, which would be the most critical regions in terms of stress if there were no glass microspheres. Consequentially, the failure pattern changes from adhesive to cohesive.

Finally, as a way of testing the applicability of simple analytical methods to predict the joint’s failure load, the global yielding criterion was used. This criterion, which considers that the entirety of the overlap is transferring the load, was chosen, since it is applicable for short overlaps and when the substrates do not deform plastically, which was the case for the SLJ geometries being studied [20].

To make use of this criterion, the following equation was used [20]:

*P*_GY_ = *τ_y_*·*b*·*l* (1)

where *P*_GY_ is the adhesive’s failure load as a consequence of its global yielding, *τ_y_* is the adhesive’s yield strength, *b* is the bonded joint’s width, and *l* corresponds to the overlap length [20].

For the SLJs being studied, the bonded joints have a width of 25 mm and an overlap length of 12.5 mm. Taking this into account, and knowing that the neat adhesives’ yield strengths were 30.9 ± 0.9 MPa and 33.9 ± 0.2 MPa, for Adhesive A and Adhesive B, respectively, Table 2 compares the experimental and the analytical values of the failure load for neat Adhesive A and neat Adhesive B. The yield strength values for each adhesive were determined using bulk tensile testing specimens in a parallel work. 

By analyzing the results represented in Table 2, it is possible to conclude that the global yielding criterion provides a satisfactory estimation of the bonded joint’s failure load, since the errors associated to the values calculated with this equation are relatively small. Furthermore, it is also possible to observe that for Adhesive A, the analytical prediction is higher than the experimental value.

In fact, one can conclude that, by calculating the shear strengths of the SLJs for Adhesive A and Adhesive B, these values were similar to the tensile strength values that were obtained during the adhesive characterization process. Furthermore, the shear strengths for some of the SLJ configurations revealed shear strength values which were slightly higher than those associated with the tensile strength. This could have been the consequence of the different strain rates being applied for the SLJs and for the bulk specimens. Even though a quasi-static speed of 1 mm·min^−1^ was applied for both cases, the length of adhesive which was being displaced was much smaller during the SLJ testing, since the very thin bonded region had an overlap length of 12.5 mm, while the thicker bulk specimen had a length of 150 mm. For this reason, the deformation, in terms of strain, that was applied to the SLJs was much higher, which resulted in higher strain rates. Due to the fact that the two adhesives are designed for automotive applications, their mechanical performance is optimized for impact applications, showing improved shear strengths for higher strain rates; as Borges et al. [21] also reported in an experimental study on the influence of loading rate on crash resistant adhesives. Taking this into account, it is to be expected that the shear strength will be higher than the tensile strengths obtained during bulk specimen testing.

### 3.2. Single Lap Joint Tests with Mild Steel Substrates

**Figure 12:**
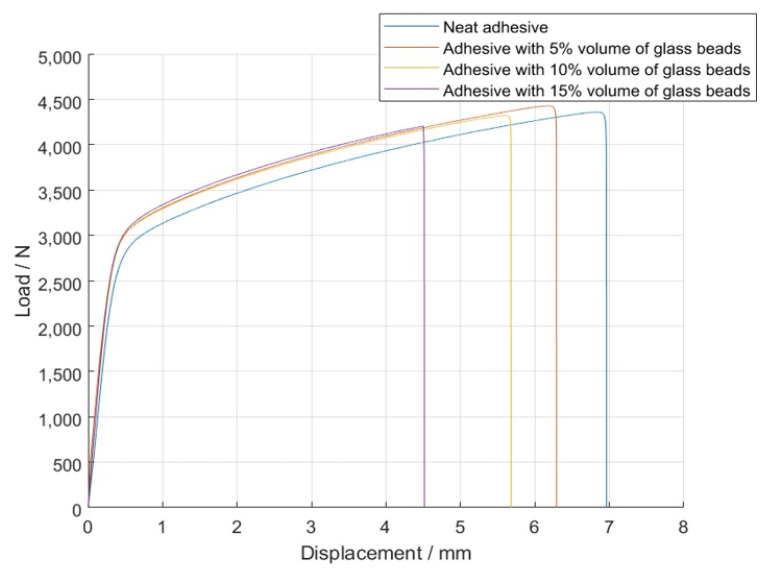
Representative *P-δ* curves for the single lap joint tests of Adhesive A with neat adhesive, and the addition of 5%, 10%, and 15% volume of hollow glass beads.

The *P-δ* curves obtained for the single lap joint specimens with mild steel were those that follow in Figure 12, for Adhesive A at quasi-static conditions.

**Figure 13:**
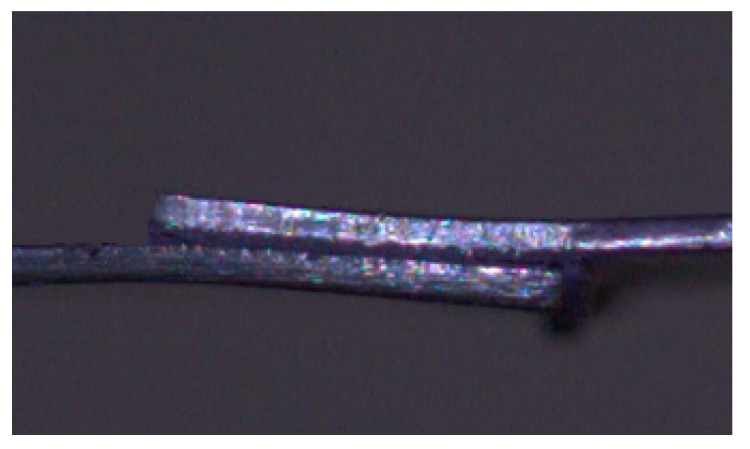
Representative image of the plastic deformation to which the mild steel substrates were subjected during the SLJ tests.

The curves represented in Figure 12 clearly show that, after reaching a load value between 2500 and 3000 N, it is the substrate, instead of the adhesive, that controlled the performance of the single lap joint specimens tested. This occurred due to the fact that the adherends were, not only thin (with a thickness of 0.7 mm), but also made of mild steel, which allowed them to deform plastically. This phenomenon can be verified by observing the deformation of the substrates throughout the SLJs tests, as shown in Figure 13.

By analyzing the *P*-*δ* curves, one can notice that the area below them generally decreases with increasing amounts of glass particles in the adhesive. Table 3 highlights the decrease in percentage of the area below these curves for the different amounts of glass beads tested. This trend is in line with the decrease of the adhesive’s ductility with the increasing volume of glass beads that was observed in a previous work by the authors.

**Figure 14:**
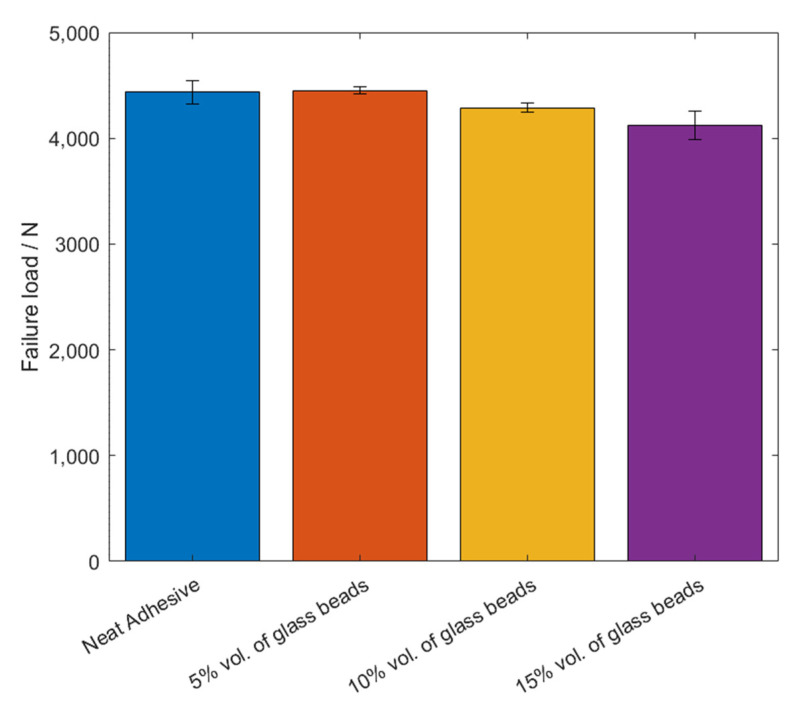
The failure load values, *P_max_*, of Adhesive A for the different percentage volumes of hollow glass beads added.

**Figure 15:**
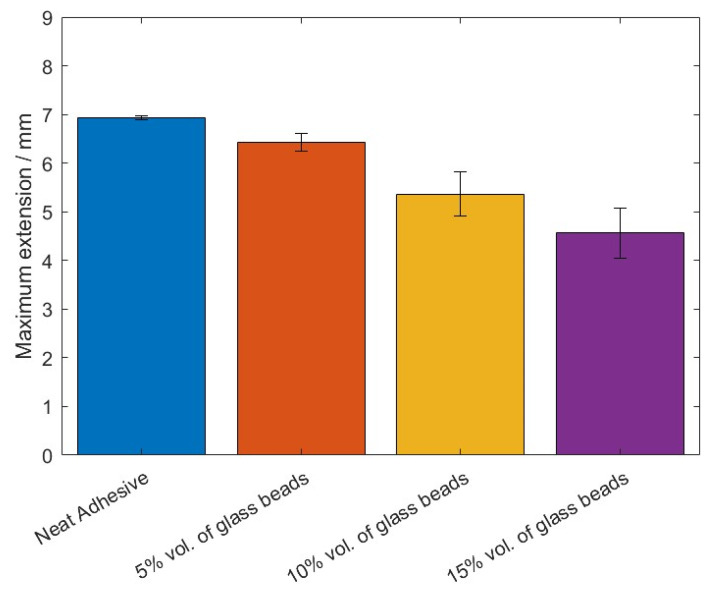
The maximum extension values, *δ_max_*, of Adhesive A for the different percentage volumes of hollow glass beads added.

The values of the failure load, *P_max_*, and the maximum extension, *δ_max_*, can be observed in Figure 14 and Figure 15, for the different percentage volumes of hollow glass beads added.

By taking into consideration all the experimental results that were obtained, it is possible to generally conclude that the presence of the glass microspheres in Adhesive A weakened the adhesive, since not only the area below the curves gradually was reduced, as shown in 3, but so was the maximum extension, which represents a decrease of the adhesive’s mechanical performance.

In terms of the failure load, there was no significant impact caused by the presence of the spheres, even though there was a small reduction of joint strength with increasing amounts of glass microspheres.

With the intention of comparing the results obtained experimentally with analytical methods, the methodology proposed by Adams et al. [22] to determine the strength of SLJs as a function of the overlap length and the adherend thickness was used. Considering the fact that, in this particular case, the adherend was yielding, the following equation was used:

*P*_AY_ = *σ_y_·b·t_s_/*(1 + 3*k*) (2)

where *σ_y_* is the yield strength of the adherend. For low loads and short overlaps, *k* can be assumed to be 1, resulting in:

*P*_AY_ = *σ_y_·b·t_s_*/4 (3)

For a joint with a length much larger than its thickness, such that *l/t_s_* ≥ 20, the value of *k* reduces until tending to zero, which leads to:

*P*_AY_ = *σ_y_·b·t_s_* (4)

**Figure 16:**
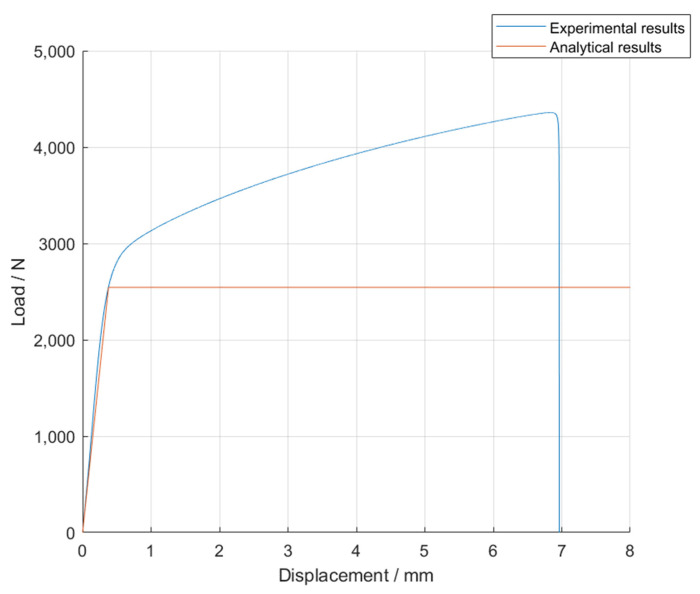
Schematic representation of the differences between the experimental and the analytical results for the single lap joints with mild steel.

Since for this case, the overlap is short, *k* can be assumed to be 1, which leads to a maximum load, *P*_AY_, of 2.54 kN. Analytically, this methodology assumes that when the adherend starts to yield, the *P-δ* curve reaches a plateau. However, and as one can observe in Figure 12, this does not happen experimentally, since the substrates, when strained beyond their yield point, require the application of additional stresses to further deform the steel; a phenomenon which is commonly referred to as the strain hardening effect. This can be observed by analyzing a schematic representation of the differences between the analytical and the experimental mechanical behavior of the bonded joint, represented in Figure 16.

**Figure 17:**
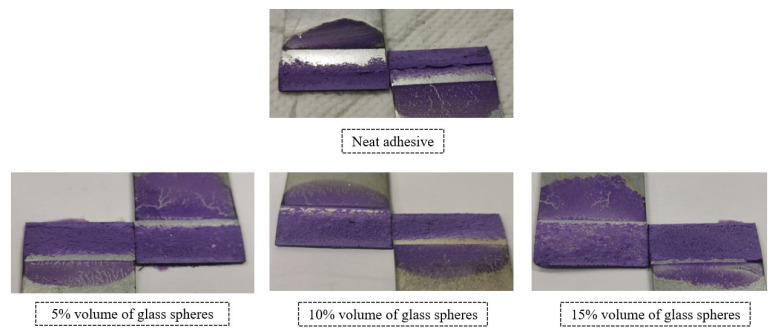
SLJ fracture surfaces of Adhesive A as a function of the amount of added hollow glass beads that were employed.

Figure 17 represents the different failure modes that were detected for the four studied cases.

By comparing the fracture surfaces exhibited in the figure above with the ones represented in Figure 7, it is possible to observe that, unlike for the single lap joint specimens with high strength steel, the failure was not cohesive for the single lap joints with mild steel substrates and neat adhesive. The reason why this happened is associated with the substrates’ plastic deformation, which increases the local stress concentrations at the ends of the bonded joint and creates critical peel stresses in those areas. The location of these stress fields causes the crack to propagate closer to the interface, which contributes to adhesive failure.

Nevertheless, the fracture surfaces show a decrease of the bonded regions without adhesive with the increasing amounts of particles. The glass microspheres introduce local stress concentrations that change the crack’s path and cause a transition from adhesive failure to a cohesive failure close to the middle of the adhesive layer.

**Figure 18:**
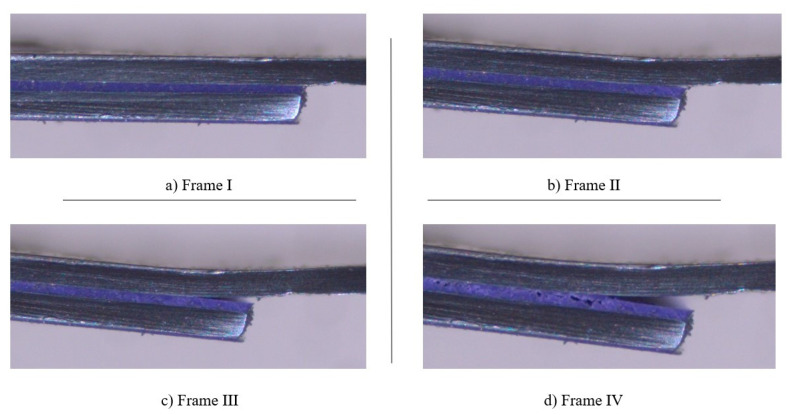
Representative frames of the different stages associated with the failure of a SLJ with neat Adhesive A.

**Figure 19:**
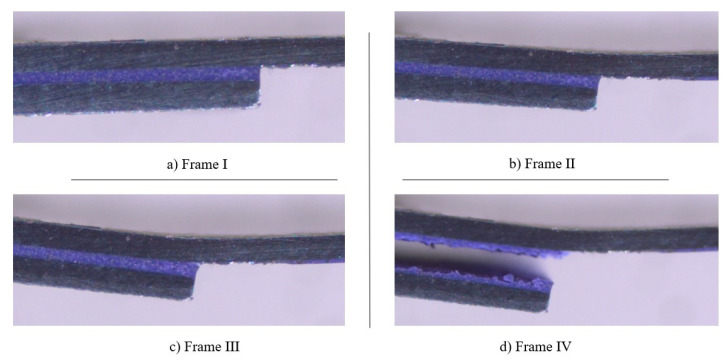
Representative frames of the different stages associated with the failure of a SLJ with the addition of glass beads to Adhesive A.

Figure 18 and Figure 19 exhibit the different stages associated to the failure of SLJs with neat adhesive and the addition of glass beads, respectively. Frames I, II, III, and IV correspond to the stages before, in the beginning, during, and at the end of the crack’s propagation, respectively.

Figure 18 shows that the crack starts to propagate in the interfacial region and continuously follows that path as the joint is loaded, as represented in frames II and III. This happens as a consequence of the substrates’ plastic deformation, which promotes very significant peel stresses in the ends of the bonded joints, turning those areas into the most critical ones, in terms of stresses. Finally, as shown in frame IV, the crack moves closer to the middle of the adhesive layer, just before the joint’s failure.

Moreover, with regards to the SLJ with Adhesive A and the addition of glass beads to the adhesive layer, Figure 19 displays a different event to the one described for neat Adhesive A. Even though there was still an interfacial crack in the initial period of its propagation, this occurred much later than in the neat adhesive. Furthermore, the crack path is noticeably shorter and, just before failure, it strayed from the interfacial zone and moved to the middle of the adhesive layer, a direct consequence of the presence of the glass beads.

**Figure 20:**
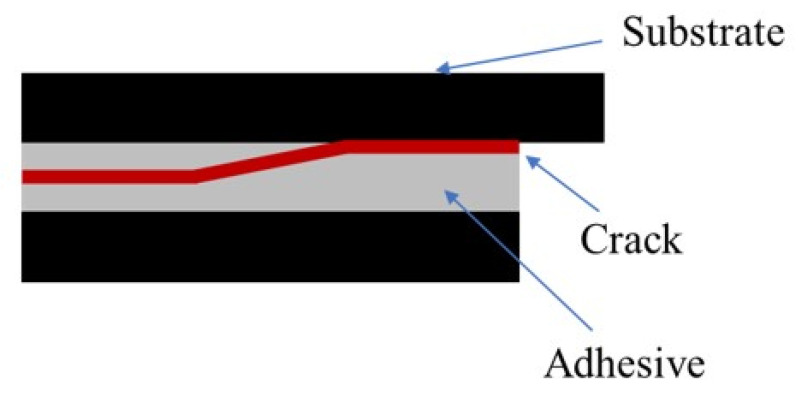
Representative scheme of the crack propagation path of a SLJ with neat Adhesive A.

**Figure 21:**
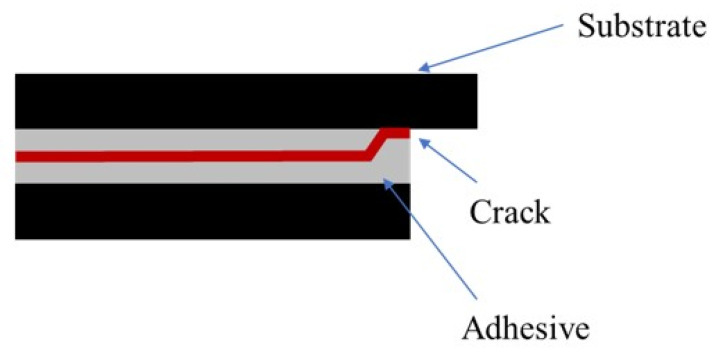
Representative scheme of the crack propagation path of a SLJ with the addition of glass beads to Adhesive A.

Figure 20 and Figure 21 exhibit representative schemes of the path followed by the crack with neat adhesive and with the addition of glass beads, respectively.

**Figure 22:**
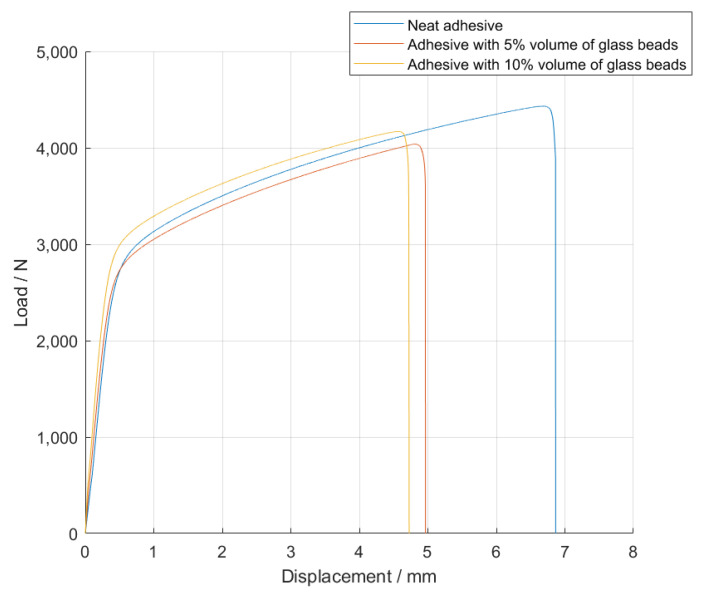
Representative *P-δ* curves for the single lap joint tests of Adhesive B with neat adhesive and 5% and 10% volume of hollow glass beads.

Regarding the SLJs tests for Adhesive B, the *P-δ* curves recorded at quasi-static conditions are represented in Figure 22.

In a similar way to what was stated regarding SLJs with Adhesive A, the *P-δ* curves represented in Figure 22 show that the substrates were also deformed plastically. Moreover, it is evident that the area below these curves was decreased by introducing glass particles into the adhesive layer, as one can confirm in Table 4.

**Figure 23:**
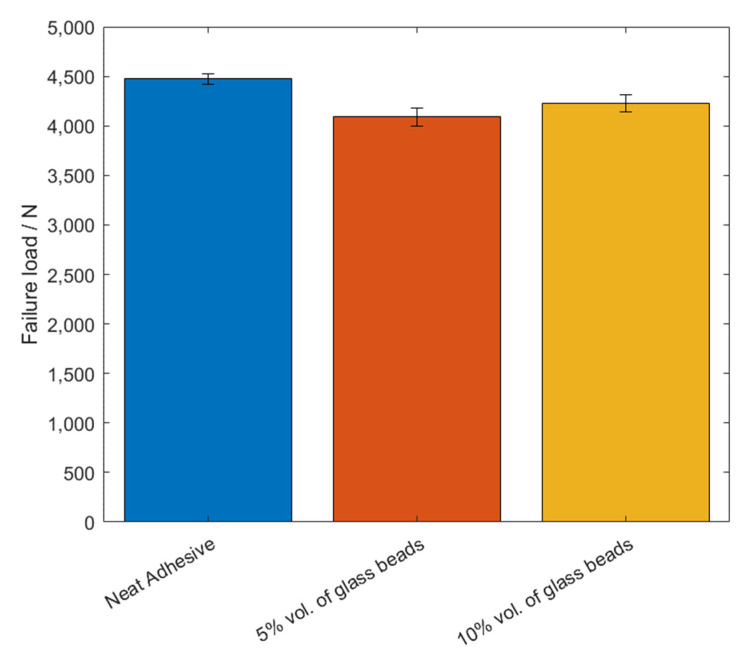
The failure load values, *P_max_*, of Adhesive B for the different percentage volumes of hollow glass beads added.

**Figure 24:**
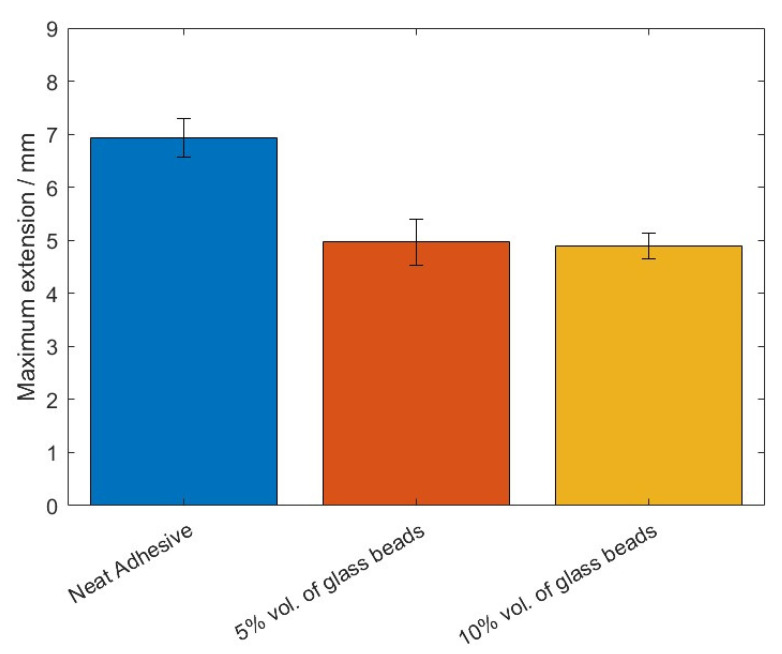
The maximum extension values, *δ_max_*, of Adhesive B for the different percentage volumes of hollow glass beads added.

The mean values of the failure load, *P_max_*, and the maximum extension, *δ_max_*, are shown in Figure 23 and Figure 24, for the different percentage volumes of hollow glass beads added.

Regarding the results of testing of the SLJ specimens with mild steel and Adhesive B, one can conclude that, by adding glass beads to the adhesive, the joint’s performance declined; since in the mechanical characterization described in a previous work by the authors the adhesive’s properties were negatively affected with the introduction of glass microspheres into the adhesive layer. Again, the failure load values did not significantly vary by adding glass beads to the adhesive. Moreover, the maximum extension decreased, while the Young’ modulus increased, which indicates that the adhesive became more brittle due to the presence of particles within the matrix.

**Figure 25:**
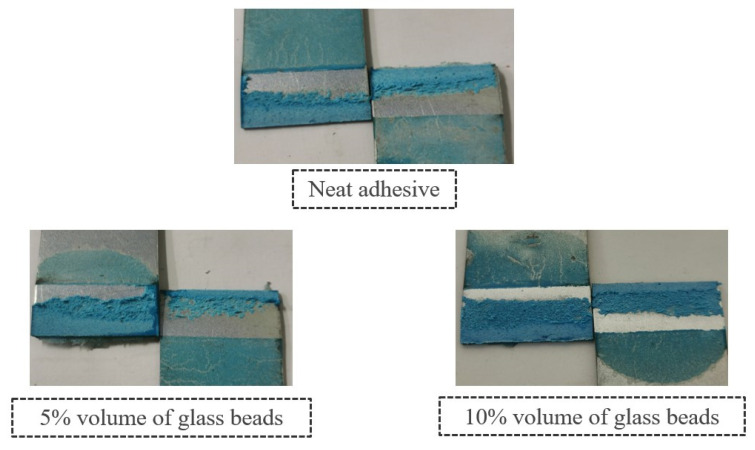
SLJs fracture surfaces of Adhesive B as a function of the amount of added hollow glass beads that were employed.

In order to support the experimental results that were shown previously, it is also relevant to report the representative failure patterns of each of the SLJ configurations. With this in mind, Figure 25 exhibits the fracture surfaces observed for the different volumes of hollow glass beads.

Unlike what occurred with Adhesive A, the presence of the spheres did not result in a transition from adhesive to cohesive failure. However, the area of the regions without traces of adhesive at the ends of the bonded joints seemed to be decreased. Therefore, it is reasonable to infer that, if the number of glass beads were further increased, then the areas without adhesive would gradually decrease until cohesive failure was reached; similarly to what was reported for Adhesive A.

**Figure 26:**
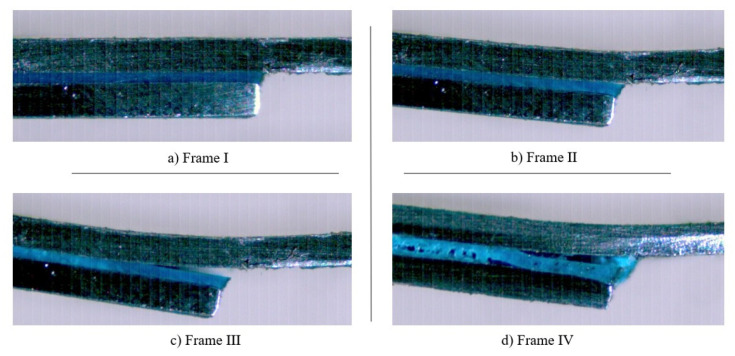
Representative frames of the different stages associated with the failure of a SLJ with Adhesive B.

For Adhesive B, and similarly to what was done for Adhesive A, the SLJs tests were recorded using a high-speed camera. In accordance with the fracture surfaces displayed in Figure 25, the addition of glass beads to the adhesive layer did not result in a change of the crack propagation path. In fact, the failure pattern reported with neat adhesive and with the addition of glass particles was rather similar, as can be observed in Figure 26. Frames I, II, III, and IV correspond to the stages before, in the beginning, during, and at the end of the crack propagation, respectively.

Observing the representative frames displayed in Figure 26 allows us to conclude that the failure pattern of the SLJs with Adhesive B closely resembles the failure pattern observed in the SLJs with neat Adhesive A. Again, the crack starts to propagate near the interface and continually follows that path. Finally, just before failing, its direction changes, moving closer to the middle of the adhesive. With that said, the representative scheme presented in Figure 20 is also applicable for reproducing the crack propagation path for the SLJs with Adhesive B.

### 3.3. SEM Analysis

**Figure 27:**
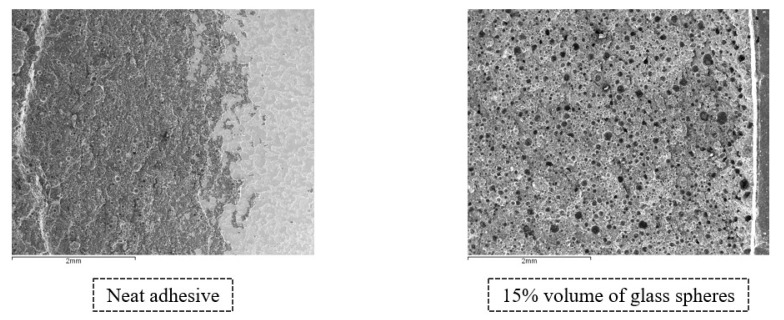
Fracture surfaces of SLJs of Adhesive A with neat adhesive and adhesive with 15% volume of glass beads added, obtained through secondary electron analysis.

Starting with the analysis of the fracture surfaces of the SLJs with Adhesive A, Figure 27 displays a comparison between the transition regions (from the adhesive to the end of the bonded joint) in the SLJs with neat adhesive and 15% volume of glass beads; the two most extreme configurations.

Figure 27 corroborates what was concluded from visually examining the bonded regions represented in Figure 17, since it is possible to verify that the substrate areas covered by adhesive have a clear increase, from the neat adhesive configuration to the one with 15% volume of glass beads; resulting from an evolution from adhesive to a cohesive failure.

**Figure 28:**
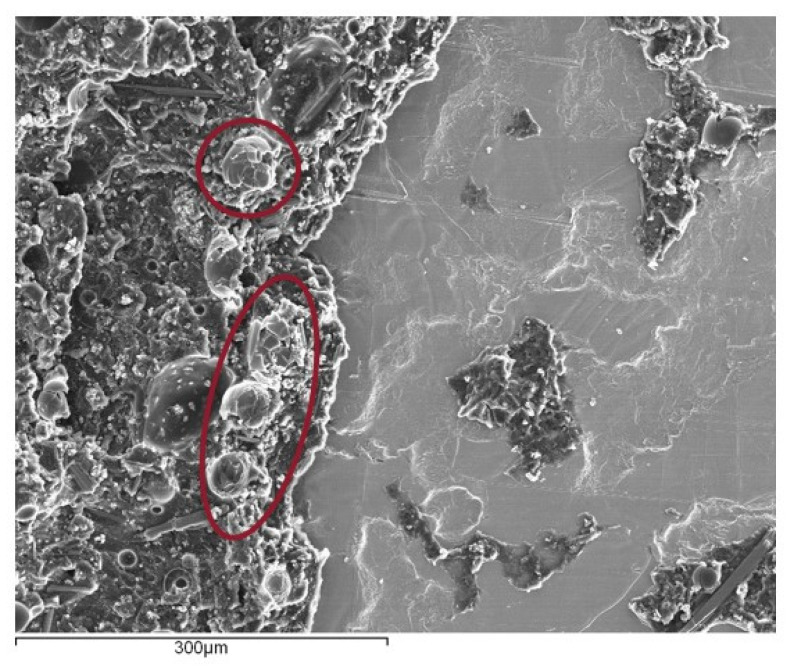
Fracture surface of a SLJ specimen with Adhesive A and 10% volume of glass beads added, with a focus on the region around the glass beads (circled in red), obtained through secondary electron analysis.

Furthermore, in Figure 28, it is possible to observe that, although adhesion between the beads and the matrix is good, a significant number of glass beads show a high level of damage. The stress fields that form in the regions around these beads result in localized cracks around the particles. These cracks will eventually bridge the gaps between the particles and coalesce, eventually leading to complete breakage of the adhesive.

**Figure 29:**
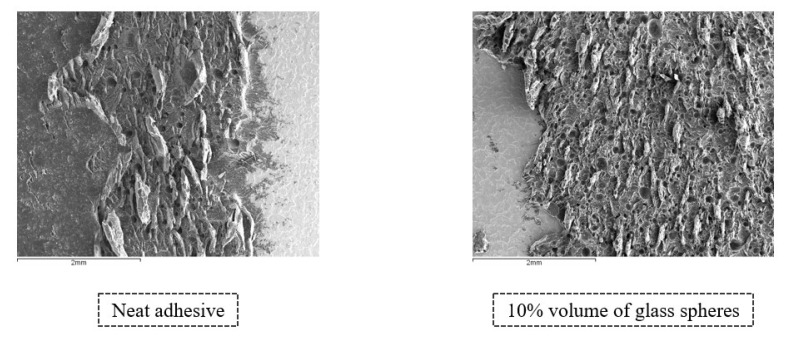
Fracture surfaces of SLJs with neat Adhesive B and adhesive with 10% volume of glass beads added, obtained through secondary electron analysis.

With regards to the SLJs bonded with Adhesive B, Figure 29 represents the transition regions in the SLJs with neat adhesive and a 10% volume of glass beads, which are, for this adhesive, the most extreme cases, in terms of the amounts of glass particles.

As one can conclude from observing Figure 29, the addition of glass beads does not lead to a complete cohesive failure for Adhesive B, and there is no evidence of cracking around the glass particles, as was the case for Adhesive A. However, the area of the regions which are not covered by the adhesive seems to decrease, in accordance with the conclusions from Figure 25.

**Figure 30:**
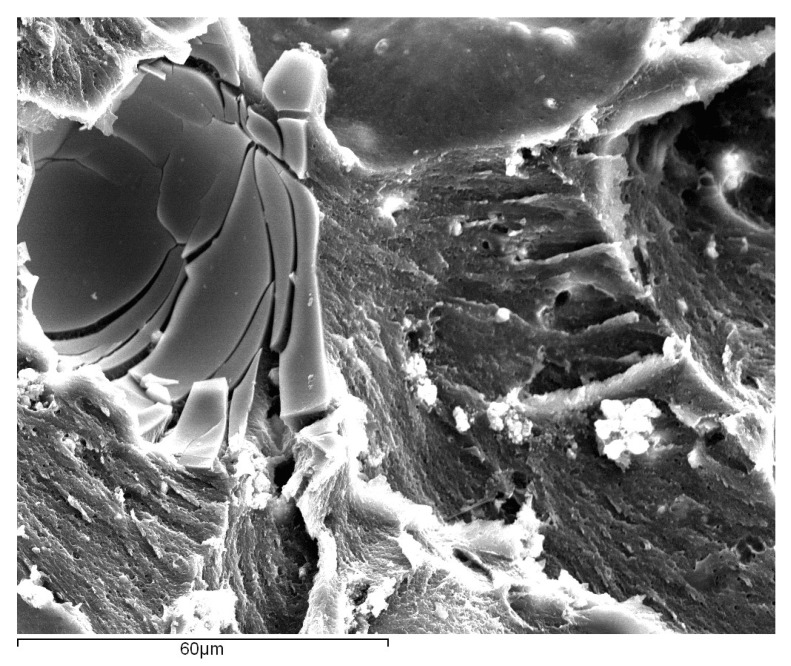
Fracture surface of a SLJ specimen with Adhesive B and a 10% volume of glass beads added, with a focus on the region around the glass beads, obtained through secondary electron analysis.

Finally, the case of the SLJ specimens with Adhesive B and a 10% volume of glass beads were also evaluated in terms of their damage state, Figure 30.

Again, the glass beads revealed a good level of adhesion to the adhesive matrix, and a significant number of them were found to be completely shattered. This indicates that the glass beads behaved similarly to what was reported for the SLJ specimens with Adhesive A; with the formation of local stress concentrations, which have the consequence of weakening the adhesive’s matrix.

### 3.4. Comparison between the Two Adhesives

As a way of comparing the two presented adhesives, the *P-δ* curves, as well as the respective fracture surfaces, associated with the SLJs with high strength steel and mild steel substrates will be displayed.

One should note that, since neat Adhesive A already had a 5% volume of glass beads, direct comparisons can only be made between neat Adhesive A and Adhesive B with 5% volume of glass beads added, and Adhesive A with 5% volume of glass beads added and Adhesive B with 10% volume of glass beads added, which are the two comparable cases.

#### 3.4.1. Single Lap Joint Tests with High Strength Steel Substrates

**Figure 31:**
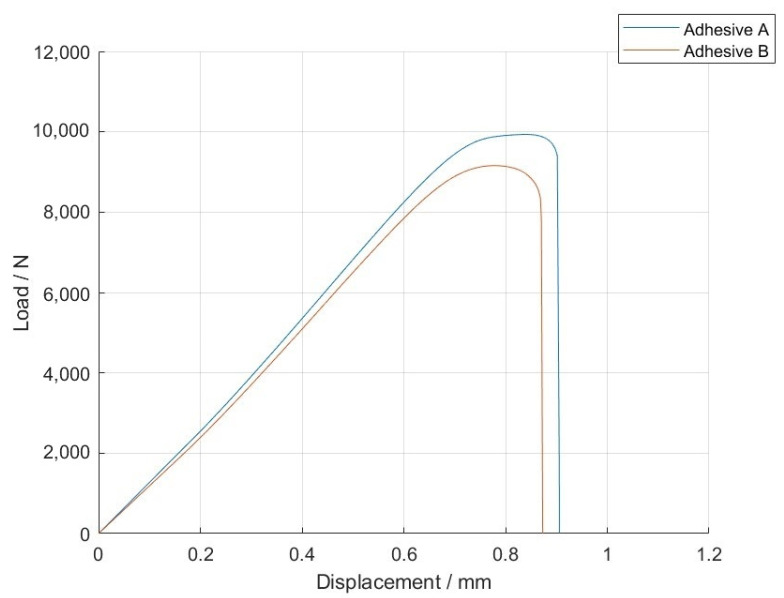
Representative *P*-*δ* curves for the single lap joint tests of adhesives with 5% volume of glass beads.

**Figure 32:**
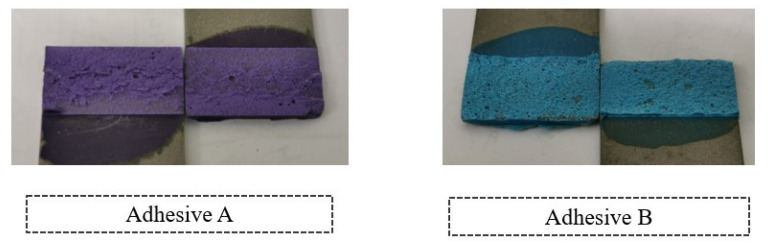
SLJs fracture surfaces of adhesives with 5% volume of glass beads.

For the adhesives with 5% volume of glass beads, the *P-δ* curves and the fracture surfaces of each adhesive are presented in Figure 31 and Figure 32.

**Figure 33:**
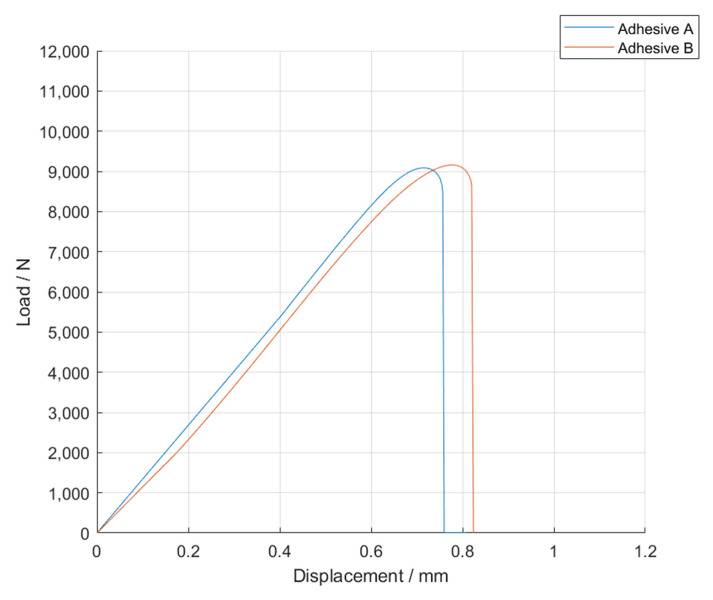
Representative *P-δ* curves for the single lap joint tests of adhesives with 10% volume of glass beads.

**Figure 34:**
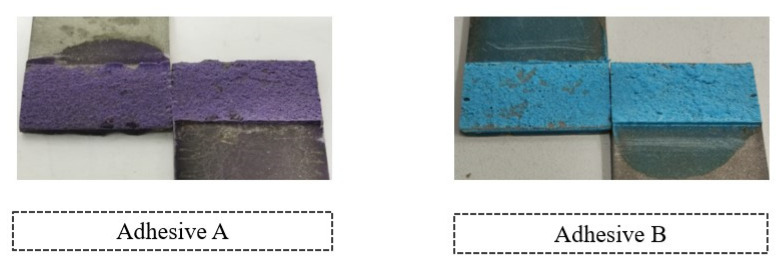
SLJs fracture surfaces of adhesives with 10% volume of glass beads.

For adhesives with 10% volume of glass beads, the *P-δ* curves and the fracture surfaces of each adhesive are presented in Figure 33 and Figure 34.

Adhesive A is shown to have had a slightly better mechanical performance than Adhesive B for 5% volume of glass beads. For 10% volume of glass beads, Adhesive B showed a better mechanical behavior, even though both show very similar results. With regards to the SLJs fracture surfaces, the failure mode was always fully cohesive in nature, indicating that the glass beads slightly weakened the adhesive layer and ensured that the crack travels through the middle of the adhesive layer thickness.

#### 3.4.2. Single Lap Joint Tests with Mild Steel Substrates

**Figure 35:**
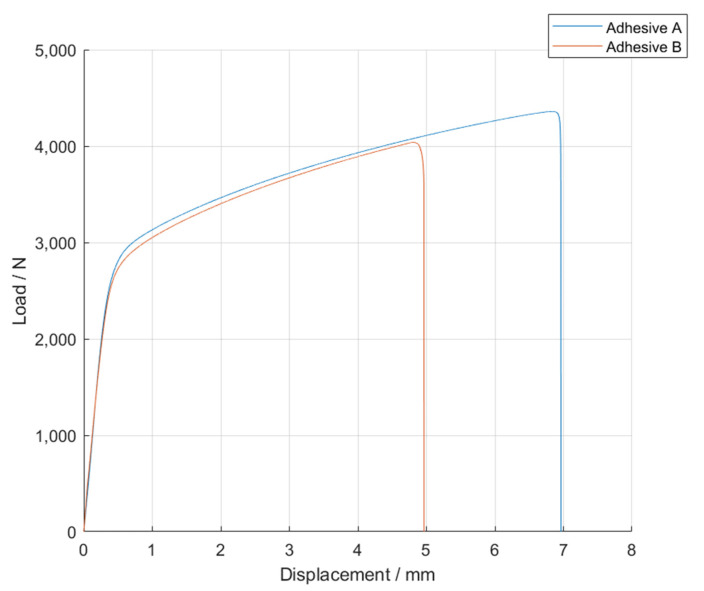
Representative *P-δ* curves for the single lap joint tests of adhesives with 5% volume of glass beads.

**Figure 36:**
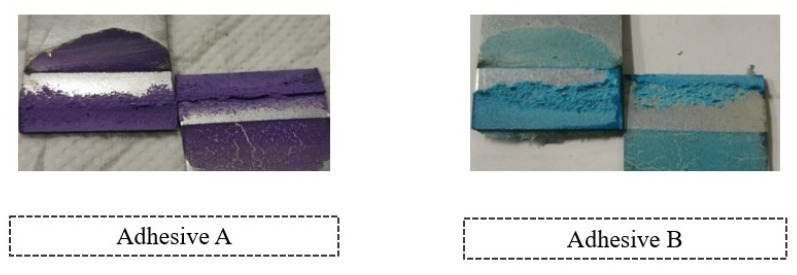
SLJs fracture surfaces of adhesives with 5% volume of glass beads.

For the adhesives with 5% volume of glass beads, the *P-δ* curves and the fracture surfaces of each adhesive are presented in Figure 35 and Figure 36.

**Figure 37:**
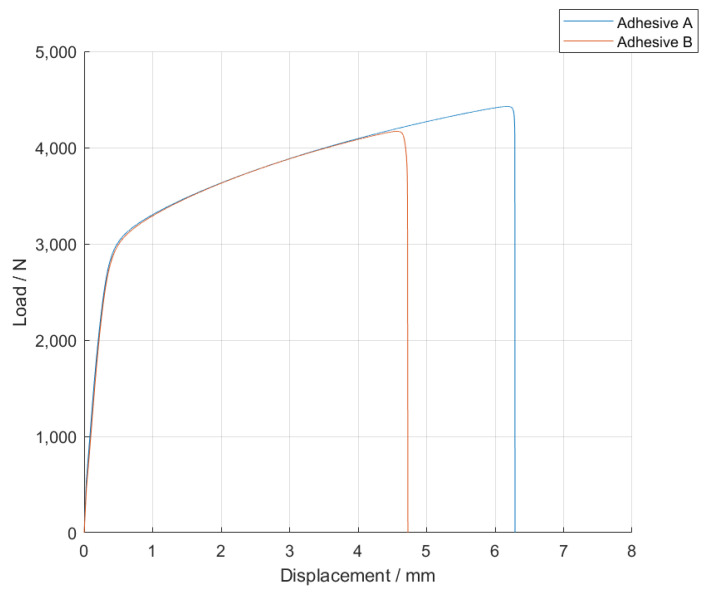
Representative *P-δ* curves for the single lap joint tests of adhesives with 10% volume of glass beads.

**Figure 38:**
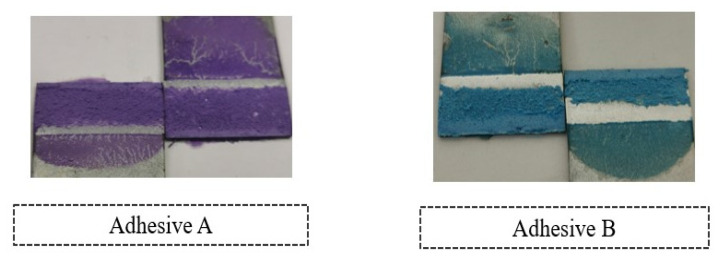
SLJs fracture surfaces of adhesives with 10% volume of glass beads.

For the adhesives with 10% volume of glass beads, the *P-δ* curves and the fracture surfaces of each adhesive are presented in Figure 37 and Figure 38.

The results represented above establish that Adhesive A had a better mechanical performance, both for 5% and 10% volume of glass beads. One can also see that the difference between the two adhesives decreased slightly with a 10% volume of glass beads.

Both adhesives show an adhesive type of failure for the 5% volume of glass beads, indicating that the level of adhesion of the adhesive to the substrates was somewhat lower than the internal cohesive strength of the adhesive. However, by adding a further 5% volume of glass beads, the failure mode noticeably changed from adhesive to cohesive, for Adhesive A. As stated previously, this happens as a consequence of the presence of the hollow glass microspheres, which introduce local stress fields into the adhesive that surrounds them. This added level of stress effectively weakens the bulk of the adhesive layer, forcing cracks to propagate closer to the middle of the adhesive layer thickness, rather than through its initially weaker interfacial region. Furthermore, for Adhesive B, even though the failure mode is still adhesive in nature, it is possible to observe that the bonded regions without adhesive clearly decreased when increasing the number of glass beads, which happens as a result of the same phenomenon as reported above.

To sum up, Adhesive A would be a better solution for bonding mild steel, due to the fact that it, not only provides a better mechanical performance than Adhesive B, but it also shows cohesive failure for 10% volume of glass beads, which is often the preferable failure mode for many industrial engineering applications.

## 4. Conclusions

The addition of hollow glass beads to an adhesive layer was investigated, with a special focus on the way this process affected the joint’s strength and the corresponding failure mechanism. To that aim, single lap joint specimens with mild steel and high strength steel substrates were tensile tested with different amounts of glass beads. The tensile tests allowed determining the mechanical performance of the joints under study, and the corresponding fracture surfaces were analyzed. All tests were performed at a quasi-static test rate.

The main conclusions extracted from this study are the following:

- Testing of SLJs with high strength steel substrates showed that adding glass beads to the adhesive generally worsened the mechanical performance of the bonded joints. This was found to be true for both adhesives. Moreover, for Adhesive B, the addition of particles to the adhesive layer forced the fracture mode to evolve from adhesive to cohesive failure.
- For both adhesives, the SLJs specimens with mild steel substrates were found to be more susceptible to a reduction in strength as a result of introduction of hollow glass spheres to the adhesive. Doing so led the crack to propagate in the middle of the adhesive, rather than in the interfacial region, which caused the failure mode to evolve from an adhesive failure to a cohesive one.
- For Adhesive A, the addition of hollow glass spheres to the adhesive led the crack to propagate in the middle of the adhesive layer rather than in the interfacial region, which forced the failure mode to evolve from an adhesive failure to a cohesive one. This is a novel effect, which is of value for some industries which prefer to avoid failure near the interface, even at the cost of slightly lowered joint performance. For Adhesive B, even though the area of the regions without adhesive at the ends of the bonded joints decreased, the failure did not evolve from adhesive to cohesive.
- The hollow glass beads do not function as tougheners of an adhesive and joint, since they do not contribute to the creation of damage mechanisms within the adhesive. Oppositely, they create local stress concentrations in their periphery, which weaken the adhesive’s mechanical performance.

## Figures and Tables

**Table 1:** Main properties of the hollow glass beads, provided by the supplier.

Property	-
Density/g·cc^−1^	0.37
Color	White, powdery
Composition	Soda-lime-borosilicateglass
Median particle diameter/µm	45
Strength/MPa	20.6

**Table 2:** Comparison between the experimental and analytically predicted values of the failure load for neat Adhesive A and neat Adhesive B.

Adhesive	Experimental Result (N)	Analytical Result (N)	Error (%)
Adhesive A	9964.0 ± 26.4	9656.3	3.1
Adhesive B	9882.04 ± 207.5	10,593.8	7.2

**Table 3:** Decrease, in %, of the areas below the *P-δ* curves, for Adhesive A.

Added Particle Content of Glass Beads, in % Volume	Decrease (%)
0	-
5	8.4
10	16.5
15	36.4

**Table 4:** Decrease, in %, of the areas below the *P-δ* curves, for Adhesive B.

Added Particle Content of Glass Beads, in % Volume	Decrease (%)
0	-
5	32.7
10	33.5

## Data Availability

Data is contained within the article.
